# Bilateral Pulmonary Emboli on Dabigatran

**DOI:** 10.7759/cureus.15737

**Published:** 2021-06-18

**Authors:** Emily Clark, Joshua Walker, Ilya Aleksandrovskiy, Latha Ganti

**Affiliations:** 1 Emergency Medicine, Ocala Regional Medical Center, Ocala, USA; 2 Emergency Medicine, University of Central Florida College of Medicine, Orlando, USA; 3 Emergency Medicine, Envision Physician Services, Plantation, USA; 4 Emergency Medicine, HCA Healthcare Graduate Medical Education Consortium Emergency Medicine Residency Program of Greater Orlando, Orlando, USA

**Keywords:** dabigatran, acute pulmonary embolism

## Abstract

We present the case of a 44-year-old female who presented to the emergency department (ED) via emergency medical services with a chief concern of shortness of breath and was found to have bilateral pulmonary emboli (PE) while taking the direct oral anticoagulant dabigatran. The authors highlight the importance of considering PE even in patients who are on anticoagulation.

## Introduction

Pulmonary embolism (PE) is the third most common cause of death worldwide after stroke and heart attack [1]. Although the exact prevalence is unknown, the incidence of venous thromboembolism (VTE) has been estimated to be around 300,000-600,000 cases annually in the United States [2]. In parallel, longitudinal studies have shown rising annual PE incidence rates over time [3]. Untreated PE can have a mortality of up to 25% which decreases to 1%-5% when the patient is placed on anticoagulation [4]. PE most often occurs when a lower extremity deep vein thrombosis (DVT) travels to the lungs.

Many choices exist for anticoagulation. Since their development and in the general population, direct oral anticoagulants (DOACs) have become the preferred agent over vitamin K antagonists. Patients can be characterized into low-intermediate risk and high-intermediate risk based on clinical data [5]. Low-intermediate risk PE causes a right ventricular (RV) strain while high-intermediate PE causes both RV strain as well as elevated cardiac biomarkers. The treatment of submassive PE remains controversial. For patients with recurrent PE, subtherapeutic anticoagulation is the most common cause. In this case report, we describe the management of a patient who developed recurrent bilateral PE on dabigatran and the considerations for her ED management and inpatient treatment. 

## Case presentation

A 44-year-old female with past medical history significant for anxiety, seizure disorder, hypothyroidism, and multiple provoked and unprovoked DVTs and PE for which she took 75 mg oral dabigatran twice daily presented to the ED with fatigue and shortness of breath. According to the patient, she was unable to walk from her bed to the bathroom without feeling winded. She also had sharp chest pain whenever she took a deep breath with pain radiating to the right side of her neck. She presented with similar symptoms and was admitted to the hospital two days prior and was diagnosed with a DVT of the left lower extremity and PE of the right pulmonary artery. She was discharged home after one night in stable condition with a prescription for 150 mg twice daily dabigatran, which was double her previous dosage.

She had been taking her dabigatran for the last five years; however, over the past two months, she had multiple interruptions in taking it as she was instructed to stop taking dabigatran four days prior to and following a recent spinal surgery that was rescheduled multiple times. She also occasionally uses marijuana, smokes half a pack of cigarettes most days for the past 10 years, and drinks alcohol a couple of times a month. She denied oral contraceptive use.

A complete review of systems was performed and was significant for left inner thigh pain that was slowly worsening in the last few days. Vital signs obtained were oxygen saturation of 99% on room air, blood pressure 142/95 mmHg, temperature 37.2°C, pulse 98 beats per minute, and respiratory rate 18 breaths per minute. She often required deep breaths between words when talking. She was not in acute distress, however. Her physical exam was significant for tachycardia, tachypnea, and tenderness to palpation over the left thigh with no obvious deformities, swelling, or rashes seen.

Differential diagnosis included but was not limited to PE, acute coronary syndrome, pneumothorax, aortic dissection, pericarditis, pneumonia, pleurisy, and gastro-esophageal reflux. The initial diagnostic workup was remarkable for leukocytosis and elevated troponin (Table 1). A d-dimer was not obtained as the patient's pre-test probability of having a PE was too high, meaning we would have obtained a computed tomography angiogram (CTA) of the chest regardless.

**Table 1:** Patient's laboratory values. POC, point of care; GFR, glomerular filtration rate; BUN, blood urea nitrogen; AST, aspartate aminotransferase; ALT, alanine aminotransferase; PT, prothrombin time; APTT, activated partial thromboplastin time; WBC, white blood cell; RBC, red blood cell; Hgb, hemoglobin; Hct, hematocrit; MCV, mean corpuscular volume; MCH, mean corpuscular hemoglobin; MCHC, mean cell hemoglobin concentration; RDW, red cell distribution width; MPV, mean platelet volume

Chemistry	
POC Glucose (70-110)	100
Magnesium (1.8-2.5 mg/dL)	2
POC Troponin I (0.00-0.07 ng/mL)	0.19 H
Troponin I (0.00-0.034 ng/mL)	0.211 H
Sodium (136-145 mmol/L)	137
Potassium (3.5-5.1 mmol/L)	3.8
Chloride (98-107 mmol/L)	106
Carbon dioxide (22-34 mmol/L)	25
BUN (7-18 mg/dL)	12
Creatinine (0.6-1.3 mg/dL)	0.7
Est GFR (Non-Af Amer) (>60 mL/min)	>60
BUN/Creatinine ratio (12.0-20.0 ratio)	16.3
Glucose (70-110 mg/dL)	104
Calcium (8.8-10.5 mg/dL)	9.1
Total bilirubin (0.2-1.5 mg/dL)	0.5
Direct bilirubin (0.0-0.5 mg/dL)	0
AST (12-37 IU/L)	26
ALT (4-35 Unit/L)	27
Alkaline phosphatase (38-126 IU/L)	70
Total protein (6.4-8.2 g/dL)	6.7
Albumin (3.4-5.0 g/dL)	3.8
Albumin/Globulin ratio (1.1-2.0 ratio)	1.3
Coagulation	
PT (9.4-12.5 s)	11.9
INR (0.8-1.1)	1.03
APTT (25.1-36.5 s)	27.4
Hematology	
WBC (3.7-11.0 thou/mm3)	13.0 H
RBC (3.40-5.10 m/mcL)	4.49
Hgb (11.0-15.4 g/dL)	14.1
Hct (32.5-44.5%)	43.2
MCV (82.5 -98.5 fL)	96.2
MCH (26.5-35.3 pg)	31.4
MCHC (32.8-35.7 g/dL)	32.6 L
RDW (11.5-16.0%)	13.1
Plt Count (150-440 thou/mm3)	203
MPV (fL)	8.3
Neut % (Auto) (%)	57
Lymph % (Auto) (%)	35.3
Mono % (Auto) (%)	4.2
Eos % (Auto) (%)	2.5
Baso % (Auto) (%)	0.5
Absolute Neuts (auto) (2.0-7.5 thou/mcL)	7.4
Absolute Lymphs (auto) (0.7-3.1 thou/mcL)	4.6 H
Absolute Monos (auto) (0.2-0.9 thou/mcL)	0.6
Absolute Eos (auto) (0.0-0.4 thou/mcL)	0.3
Absolute Basos (auto) (0.0-0.3 thou/mcL)	0.1
Immature Gran % (%)	0.5
Immature Gran # (0.0-0.031 thou/mcL)	0.1 H

**Figure 1:**
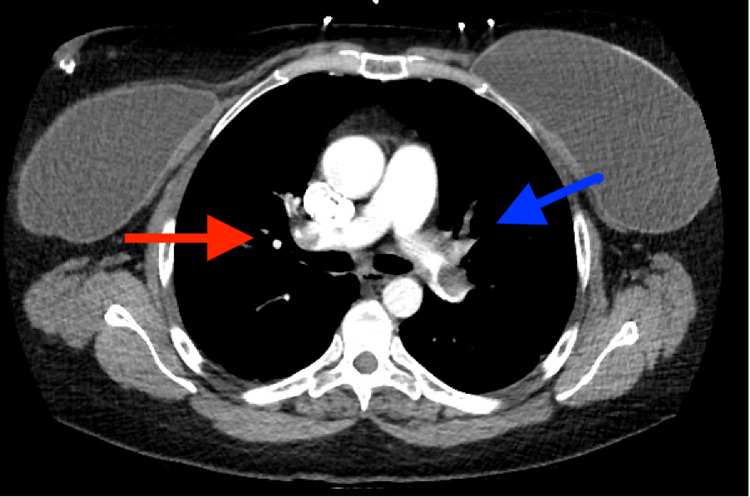
Pulmonary CTA demonstrating PE on the right (red arrow) and on the left (blue arrow). CTA, computed tomography angiogram; PE, pulmonary emboli

Computed tomography angiogram was significant for bilateral PE in the distal left main pulmonary artery extending into the left lower lobe pulmonary artery branches as well as distal right main pulmonary artery extending into the upper and lower lobes. The heart was at the upper limit of normal size (Figure 1). 

An echocardiogram demonstrated preserved left ventricular function with an ejection fraction of 55% and was significant for a right ventricular systolic pressure of 57 mmHg and left pleural effusion. Bilateral lower extremity ultrasound re-demonstrated extensive left lower extremity DVT from left common femoral, superficial femoral, popliteal, and deep calf veins. The electrocardiogram demonstrated sinus tachycardia.

In consultation with hematology, a heparin drip was initiated. The patient was subsequently admitted to the medical floor. The patient was not a candidate for ekosonic endovascular system (EKOS) due to oral anticoagulation and showed an elevated international normalized ratio (INR). She was bridged to warfarin due to the patient's preference for no longer being on dabigatran. Hematology suggested that clot formation was likely secondary to medication non-compliance (intermittent medication use) rather than the failure of dabigatran. The patient’s hospital course was complicated by multiple episodes of hematemesis as well as hematochezia that were addressed and resolved. She was ultimately bridged to warfarin and discharged home with oxygen as well as follow-up with hematology.

## Discussion

**Figure 2:**
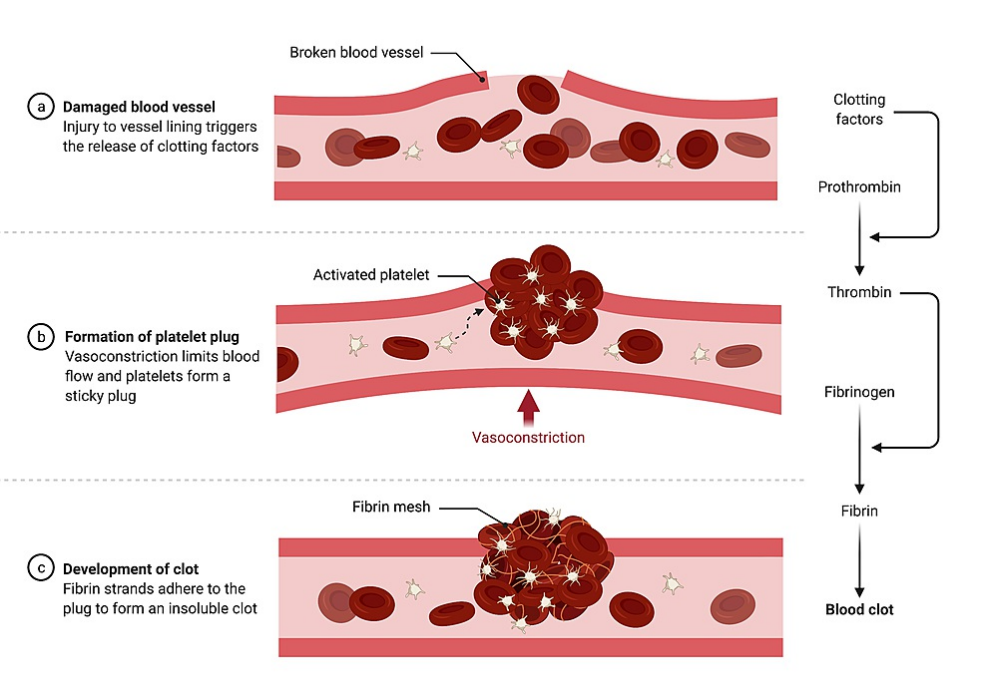
An illustration of the mechanism by which thromboemboli forms in a damaged blood vessel. Created with BioRender.com.

Pulmonary emboli (thromboemboli in the lungs) most often occur when a lower extremity DVT travels to the lungs. Lower extremity DVTs often occur secondary to stasis, hypercoagulability, or local trauma (Figure 2).

**Figure 3:**
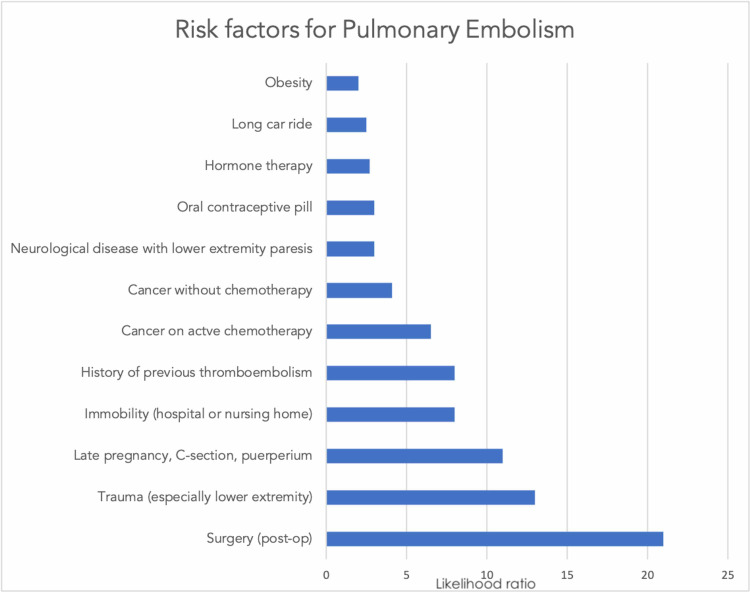
Risk factors for thromboembolism and associated likelihood ratios.

Risk factors [6] for thromboemboli such as DVT and PE are listed in Figure 3.

Dabigatran is a reversible, competitive direct thrombin inhibitor. It is able to bind and inhibit both free and clot-bound thrombin. Peak plasma concentrations of dabigatran occur within two hours of ingestion [7]. After the peak is reached, levels fall in a biphasic manner and result in a more than 70% decrease within four to six hours of ingestion. Approximately 35% of dabigatran is bound to plasma proteins and has a volume distribution of 50-70 L. In patients with normal renal function, approximately 80% of an IV dabigatran dose is excreted in urine with an elimination half-life of 12-17 hours; thus, dosing is dependent on creatinine clearance [7].

The Randomized Evaluation of Long-Term Anticoagulation (RE-LY) trial, a randomized noninferiority trial compared two doses of dabigatran with warfarin in 18,113 patients with atrial fibrillation. The trial demonstrated that at a dose of 110 mg, rates of stroke and systemic embolization were similar to warfarin, with lower rates of major hemorrhage. At a dose of 150 mg, rates of stroke and systemic embolization were lower in comparison to warfarin, however, with similar rates of major hemorrhage [7].

Dabigatran was approved by the US Food and Drug Administration for the prevention of embolic stroke in patients with non-valvular atrial fibrillation. When it was approved in 2010, it was the first new oral anticoagulant to be approved in the United States in 50 years. It is an attractive alternative to warfarin for patients and providers because it does not necessitate frequent lab monitoring due to its stable hematologic response. Also, it does not undergo CYP 450 metabolism and has few drug-drug and drug-food interactions. 

At this time, laboratory measurements to determine the therapeutic level of dabigatran are limited and often not available in the ED. For example, thromboplastin time (TT), which measures a conversion of fibrinogen to fibrin in a plasma sample is sensitive to dabigatran levels. A normal TT likely indicates subtherapeutic levels of dabigatran [8]. However, it is not commonly available in the ED. Similarly, ecarin clotting time (ECT), which measures time to the formation of the clot of plasma sample with the addition of ecarin, also demonstrates a linear response to dabigatran serum levels. More common coagulation labs such as INR, prothrombin time, and aPTT have not been shown to be sensitive markers of dabigatran levels [9].

## Conclusions

Understanding dabigatran’s mechanism of action, laboratory monitoring of therapeutic levels, and pharmacokinetics are important for emergency physicians. As in this case study, this patient presented with concerning clot burden after inconsistent dabigatran usage. It was necessary to understand how to test for appropriate therapeutic levels of dabigatran as well as timing from the last dosage of dabigatran to initiating heparin therapy. It is also important for physicians to recognize the impact of intermittently discontinuing anticoagulation. 
